# Minimally invasive plate osteosynthesis with dual plating for periprosthetic distal femoral fractures following total knee arthroplasty

**DOI:** 10.1186/s13018-021-02586-0

**Published:** 2021-07-06

**Authors:** Yong-Geun Park, Hyunseong Kang, Jung-Kook Song, Jaehwang Lee, Joseph Y. Rho, Sungwook Choi

**Affiliations:** 1 Department of Orthopaedic Surgery, Jeju National University School of Medicine, Aran 13gil 15, Jeju-si, Jeju, Self-Governing Province 63241 South Korea; 2 Department of Preventive Medicine, Jeju National University School of Medicine, Jeju, South Korea

**Keywords:** Periprosthetic distal femoral fractures, Total knee arthroplasty, Minimally invasive plate osteosynthesis, Dual locking compression plate

## Abstract

**Introduction:**

Adequate treatment for periprosthetic distal femur fractures is challenging because of various reasons, including severe osteoporosis and distal fragments that are too small or too distal. We have introduced a new surgical technique for dual plating of periprosthetic distal femur fractures following total knee arthroplasty (TKA) and determined the clinical and radiological outcomes of minimally invasive plate osteosynthesis (MIPO) with a dual locking compression plate (LCP).

**Materials and methods:**

Between January 2010 and July 2019, 18 patients [mean age, 74.8 (68–89) years; average follow-up period, 14.8 (12–43) months] underwent MIPO with distal femoral LCP laterally and proximal humeral internal locking system (PHILOS) medially for periprosthetic distal femoral fractures following TKA. The minimum follow-up was 1 year. The clinical and radiological outcomes were assessed using the modified WOMAC scores, knee range of motion, time to callus formation, time to union, and complications of malunion, nonunion, and shortening.

**Results:**

The average time to union was 18.4 weeks (range, 10–51 weeks) and to callus formation was 7.8 weeks (range, 2–14 weeks). At the 1-year follow-up, the average JLETS was 37.6 (range, 24–53), average knee ROM was 110.3° (range, 80–135°), and average varus-valgus angles of the distal femur were 3.2° (range, −2.9–10.5°). No nonunion, broken plates, or implant failure occurred. Malunion occurred in three patients.

**Conclusion:**

MIPO with dual LCP is a reliable method for stabilizing periprosthetic distal femoral fractures following TKA, with satisfactory bone union rates and low complication rates.

## Introduction

The incidence of periprosthetic distal femoral fractures following total knee arthroplasty (TKA) has been gradually increasing from 0.2 to 5.5% [1]. Risk factors include osteoporosis, female sex, elderly, rheumatoid arthritis, steroid use, and anterior femoral notching [2]. Selecting the most feasible treatment for these fractures is challenging because the severity of osteoporosis varies among patients and the amount of bone present for fixation is limited owing to the degree of comminution and distal extension of the fracture [3–5]. Periprosthetic fractures close to the implant have technical difficulties in firm fixation because of scarce bone fragments near the implant, which contributes to poor prognosis following TKA.

Various treatment options include conservative management, plate fixation, retrograde intramedullary nailing (IM), external fixation, and revisional arthroplasty [6–8]. To date, absolute stability has led to successful results [3, 7, 9]. Traditional plate fixation is highly likely to cause varus deformity and may not be feasible for very low distal femur periprosthetic fractures. Retrograde IM nailing has limited use owing to the implant design, as IM nails can pass through the intercondylar space. Distal lateral locking compression plate (LCP) used for the distal fragment increases the stability of the fracture site [10, 11]. Lateral LCP plating for distal periprosthetic femur fractures versus revision arthroplasty for extremely distal cases is still controversial [12]. Sufficient stability has been observed with the recently introduced dual plating technique [13]. Additional medial plating had medial stability against varus collapse [14], but may also cause biologic disruption and muscular damage that can adversely affect bone union and post-operative range of motion (ROM).

**Fig. 1:**
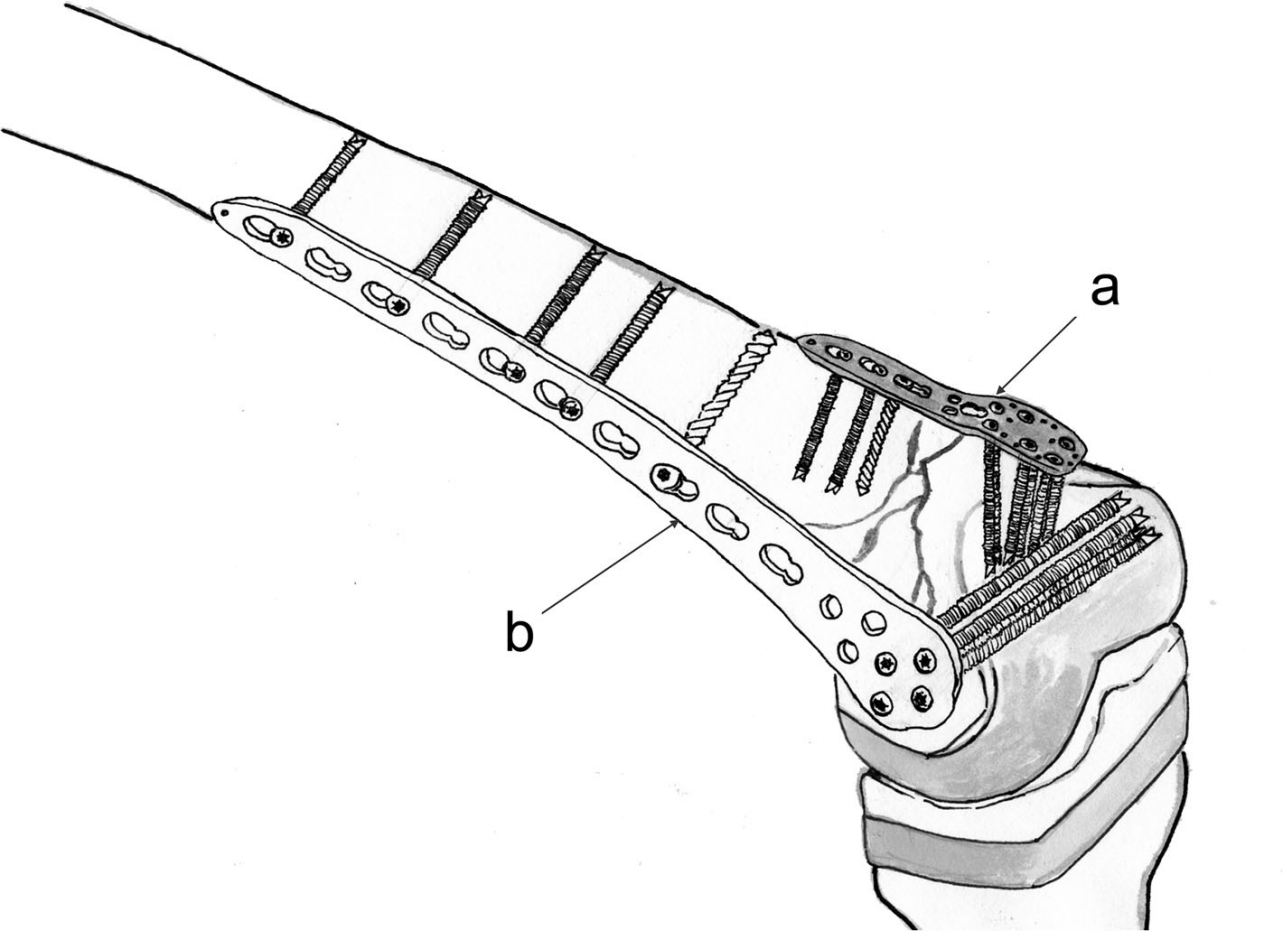
Dual plating medially with (a) PHILOS plate and laterally with (b) distal femoral LCP for the periprosthetic fracture of the distal femur

This study aimed to evaluate the effects of MIPO with dual plating to treat periprosthetic distal femoral fractures following TKA (Fig. 1).

## Materials and methods

### Patient selection

**Fig. 2:**
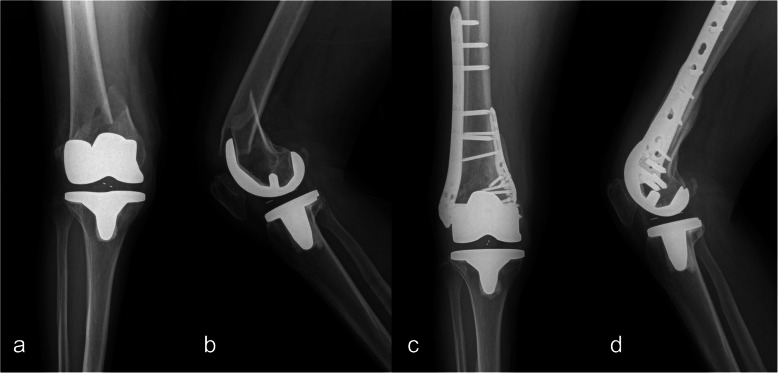
Distal femoral periprosthetic fracture after slip-down in a 68-year-old female patient. **a** Preoperative knee AP and **b** lateral radiograph show Su classification type 2 in periprosthetic fracture of the distal femur. Minimally invasive plate osteosynthesis with dual LCP was performed. Post-operative 1-year knee ROM was 125° and JLETS score was 42. **c** Post-operative knee AP and **d** lateral radiograph at 1 year show well-united fracture and satisfactory alignment

Our institutional review board approved this study. We retrospectively reviewed the medical and radiological records of 18 patients [18 women; mean age, 74.8 years (68–89 years)] with periprosthetic distal femoral fractures treated using MIPO with distal femoral LCPs (Depuy Synthes, Oberdorf, Switzerland) laterally and proximal humeral internal locking system (PHILOS, Depuy Synthes, Oberdorf, Switzerland) medially (Fig. 2), between January 2010 and July 2019. According to the Su classification system [15] of periprosthetic fractures, type 1 was noted in two, type 2 in three, and type 3 in 13 patients. The average bone mineral density (BMD) was −2.0 (−4.3–0.3). Table 1 presents the demographic characteristics.

**Table 1:** Demographic information

Patient parameter	Value
**Mean age, (years)**	74.8 ± 5.9 (68–89)
**Sex, n (%)**	
**Male**	0 (0.0)
**Female**	18 (100.0)
**Body mass index (kg/m**^**2**^**)**	24.2 ± 3.9 (16.4–29.9)
**Bone mineral density(T-score)**	−2.0 ± 1.2 (−4.3–0.3)
**ASA**^a^ **physical status class, n (%)**	
**I**	1 (5.6)
**II**	15 (83.3)
**III**	2 (11.1)
**Operative side, n (%)**	
**Left**	11 (61.1)
**Right**	7 (38.9)
**Su classification, n (%)**	
**Type I**	2 (11.1)
**Type II**	3 (16.7)
**Type III**	13 (72.2)
**Operative time (minutes)**	146.2 ± 44.8 (92–255)
**Hospitalization days (days)**	45.9 ± 32.6 (6–123)
**Follow-up (months)**	14.8 ± 10.2 (12–43)
**Time to callus formation (weeks)**	7.8 ± 3.5 (2–14)
**Time to bone union (weeks)**	18.4 ± 9.8 (10–51)
**JLETS**^b^	37.6 ± 8.5 (24–53)
**Knee ROM**^c^	110.3 ± 15.7 (80–135)
**Varus-valgus angle (degrees)**	3.2 ± 3.3 (−2.9–10.5)
**Shortening, n (%)**	12 (66.7)
**Complication, n (%)**	
**Malunion**	3 (16.7)
**Nonunion**	0 (0.0)

Note: Data presented as mean ± standard deviation unless otherwise indicated

^a^American Society of Anesthesiologists, ^b^Jeju Lower Extremity Trauma Scale (the modified WOMAC), ^c^range of motion

### Clinical and radiological evaluations

Operative time, hospitalization days, time to callus formation, time to union, post-operative complications, and clinical performance were assessed by reviewing admission and outpatient medical records. Time to bone union and callus formation were evaluated by periodic radiologic studies. Patients were followed up at 1, 3, and 6 months and at 1 and 2 years post-operatively [average follow-up period, 14.8 months (range, 12–43 months)].

Clinical evaluation, including the knee ROM and modified Western Ontario McMaster Universities Index of Osteoarthritis (WOMAC) score, was assessed at 1 year post-operatively. The modified WOMAC score (Jeju Lower Extremity Trauma Scale (JLETS)) comprises the following: pain, assessed by the visual analog scale (VAS) pain scoring system (10 points); activity score (30 points); ROM (10 points); and tenderness at the fracture site (5 points) [16].

Post-operative bone union and stability were assessed using routine anteroposterior (AP) and lateral radiographic views at each follow-up. Bone union was defined as bridging callus across the fracture site on both AP and lateral radiographs. The varus-valgus angle was determined utilizing the femur shaft axis and femoral prosthetic horizontal line connecting the medial and lateral condyles on AP radiographs [17].

Nonunion was defined as lack of healing within 6 months. Malunion was defined as a coronal deformity (varus or valgus angulation) of > 5°, sagittal deformity (anterior or posterior angulation) of > 10°, rotational deformity of > 15°, and/or shortening of > 2 cm.

The BMD of the normal hip was measured using dual-energy X-ray absorptiometry (Discovery; Hologic Inc., Bedford, MA, USA) post-operatively. The T-score of the femoral neck of the normal hip was selected as each patient’s BMD. Body mass index was measured at the time of admission.

### Surgical technique

Patients were positioned supine on the operating table under fluoroscopic guidance. The lower leg was draped from the iliac crest to the foot for intraoperative assessment of the length, rotation, and angulation. No tourniquets were utilized intraoperatively.

**Fig. 3:**
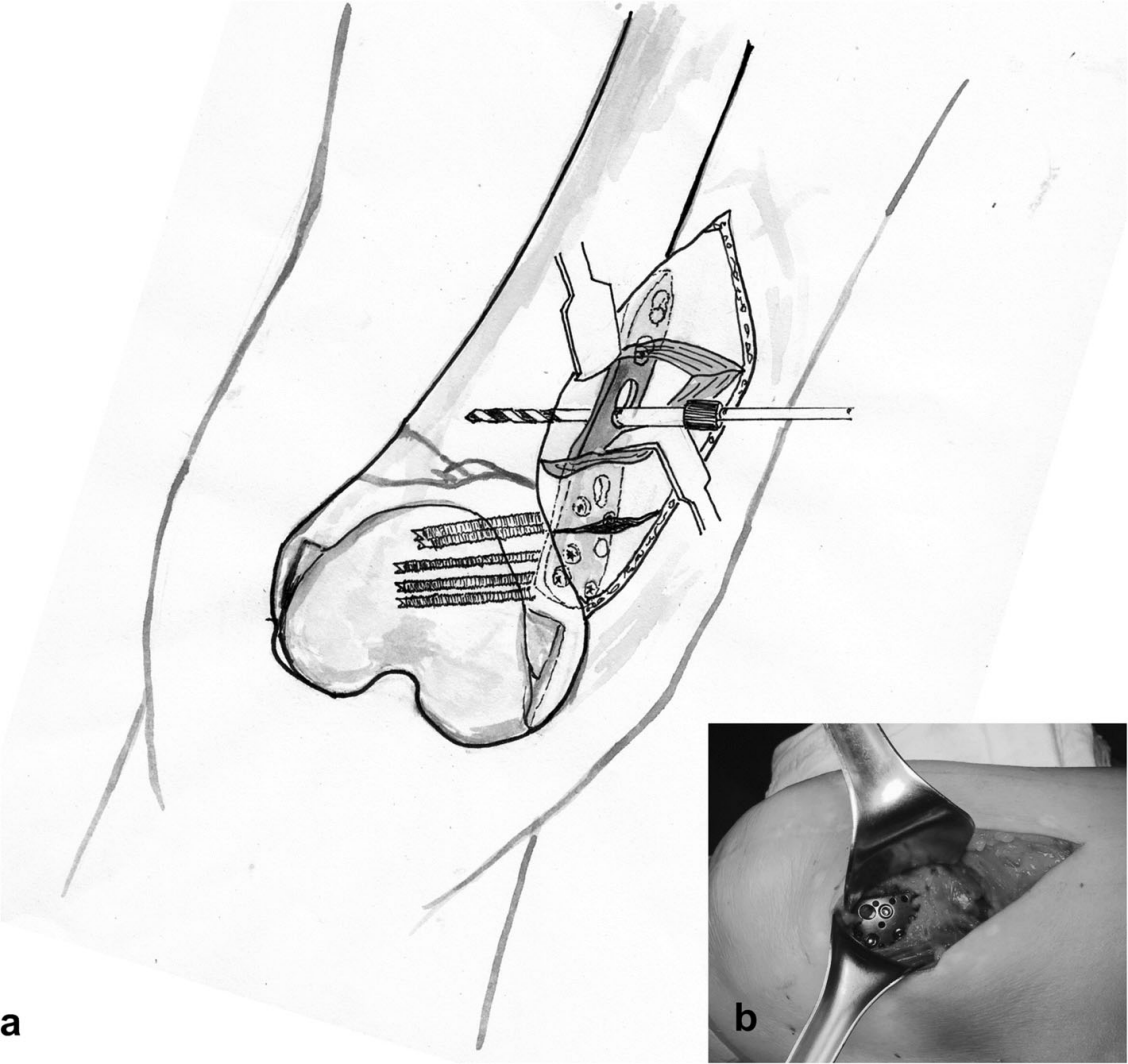
Medial plating using the PHILOS plate. The purpose of medial plating is to offer better reduction of fracture and alignment. **a** The PHILOS plate is placed at the anteromedial aspect of the femur along the contour of the distal femur and fixated with a locking screw on bone fragments. Additional muscle dissection may be needed for proximal fixation because muscle incision is made along the obliquely running muscle fibers. **b** Gross photo image of medial plating is shown in a separate box

**Fig. 4:**
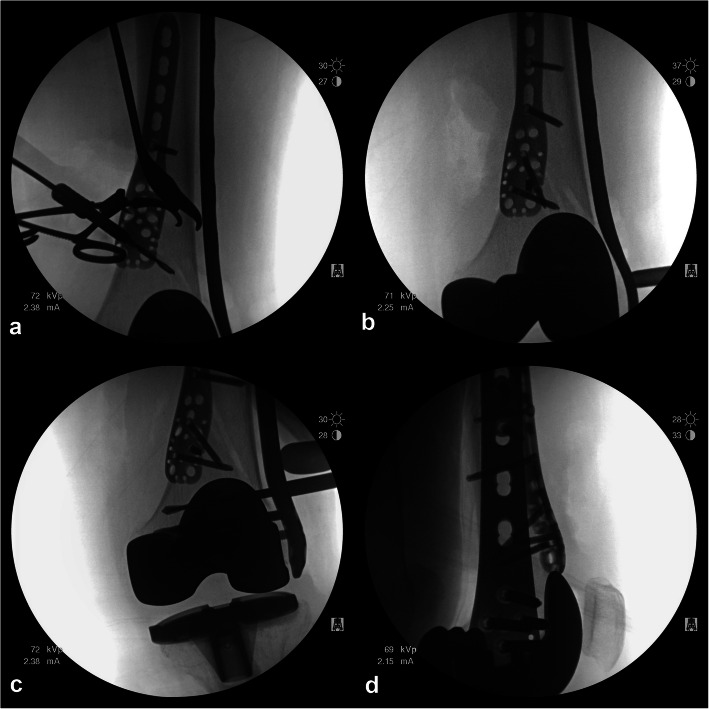
Fluoroscopic intraoperative images showing **a** provisional fixation of fracture site with bone holding forceps and **b** the placement of the lateral LCP plate following medial PHILOS plate fixation. Once the anteromedial plate fixation is done and lateral LCP plate placement is checked, **c**, **d** remaining screw fixation is finalized

A longitudinal skin incision was made anteromedially to the distal femur. Vascular cauterization was strictly performed to prevent bleeding during dissection. The fracture site was exposed after dissecting the subcutaneous tissue and deep fascia of the vastus medialis (Fig. 3). Following careful reduction with a Joker elevator to avoid periosteal damage, AP and lateral fluoroscopic views confirmed accurate reduction. On obtaining satisfactory alignment, Kirshner wires (K-wires) and bone holding forceps were placed provisionally to maintain bi-planar fluoroscopic control. The PHILOS plate was placed on the anteromedial aspect of the distal femur to correspond with the distal femoral contour, and its proximal portion was positioned at the distal medial bone fragment for optimal screw fixation. K-wires and sleeve assemblies were used to confirm the final plate placement, one in the most proximal screw hole and one in the most distal screw hole for placement in the distal femur. Medial plating was finalized by fixation with locking screws in all fixable holes (Fig. 4).

**Fig. 5:**
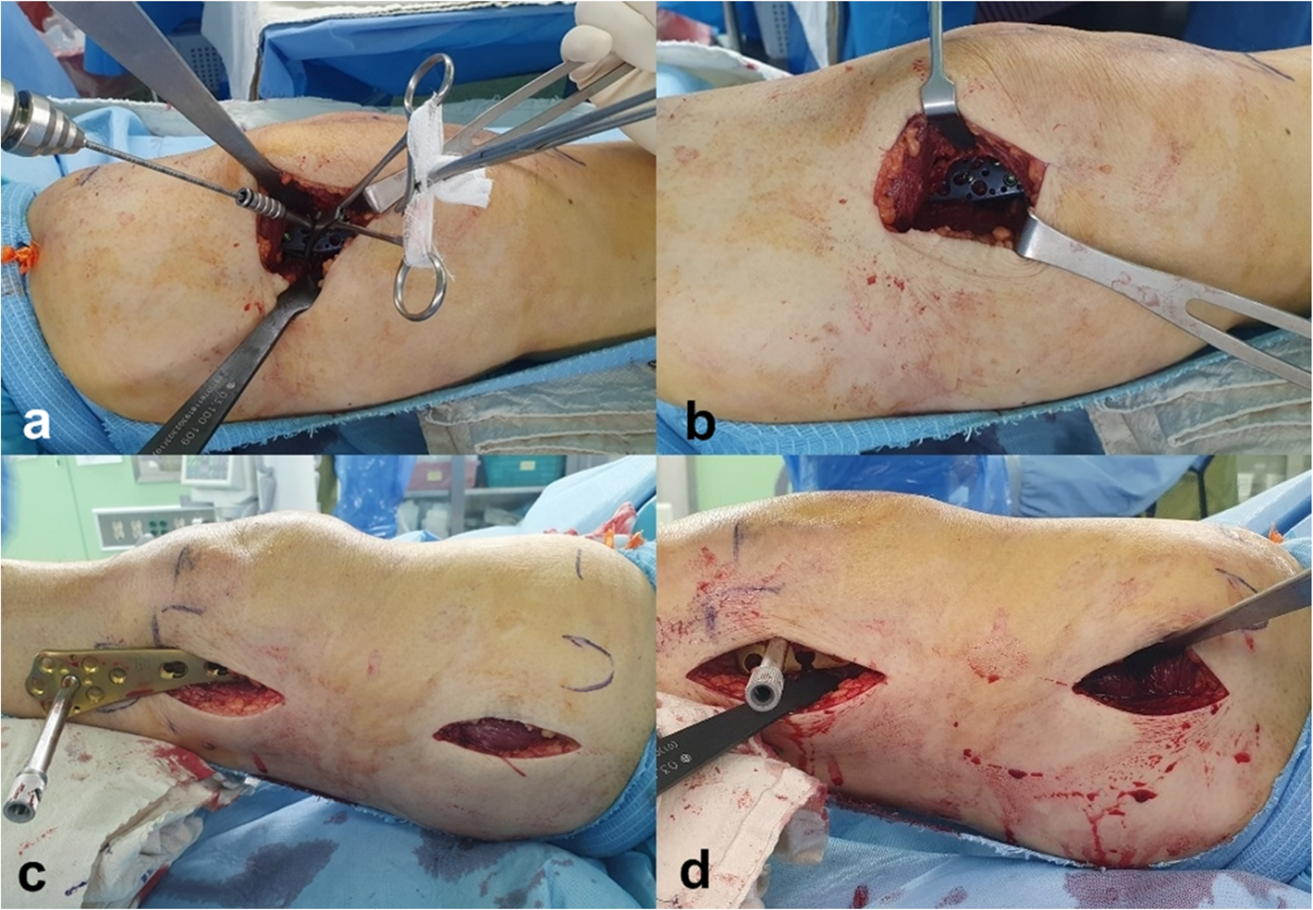
Intraoperative images showing **a**, **b** provisional fixation and medial PHILOS plate fixation through anteromedial approach and **c**, **d** fixation of lateral distal LCP plate using MIPO technique

After achieving reduction and stability to the best possible extent at the fracture site using medial plating, MIPO was performed using a distal femoral LCP for lateral plating (Fig. 5). The distal femoral LCP was selected according to the fracture location and configuration, and the plate was sufficiently long for the insertion of at least three screws at the proximal and distal mainframes. A longitudinal skin incision was made approximately 7 cm on the lateral aspect of the distal femur and the iliotibial band and vastus lateralis muscle were dissected along the direction of their fibers. A Cobb elevator was used for tunneling from the distal incision, and the LCP was inserted into the prepared tunnel. The proximal end of the plate was positioned at the center of the lateral cortex of the proximal femur without periosteal dissection. K-wires and sleeve assemblies were used to evaluate the final placement of the plate, with one in the hole for the most proximal screw and one in the hole for the most distal screw to be placed in the femur. Cortical screws were fixed to reduce the space between the plate and femoral shaft. After locking screw placement at the distal portion of the plate, at least three locking screws were inserted in the proximal portion through small discrete skin incisions. The skin was closed in layers after irrigation, and a long leg splint was applied.

### Rehabilitation protocol

Partial weight-bearing was allowed and continuous passive motion (CPM) of the knee and ankle were initiated on the second post-operative day. The CPM angle was adjusted according to each patient’s tolerance. Full weight-bearing was permitted after the pain disappeared and callus formation was radiologically confirmed.

### Statistical analysis

Statistical analysis was performed using SPSS for Windows 24.0 software package (SPSS Inc., Chicago, IL). Student’s t-test was used for descriptive statistics of mean, standard deviation, frequency, and quantitative data. Fisher’s exact chi-square test was used to evaluate radiographic outcomes. Spearman’s rho correlation coefficient analysis was used to examine the relationships between other factors. Significance was set at P < 0.05.

## Results

The average time to union was 18.4 weeks (range, 10–51 weeks) and to callus formation was 7.8 weeks (range, 2–14 weeks).

At the 1-year follow-up, the average JLETS was 37.6 (range, 24–53) and the average knee ROM was 110.3° (range, 80–135°). Fifteen patients (83.3%) showed acceptable ROM of > 100°. No patient had difficulty in daily activities and domestic duties.

Severe valgus and flexion deformity of the distal femur on the initial AP and lateral radiographs were corrected by dual LCP plate fixation. One year post-operatively, the average varus-valgus angle of the distal femur was 3.2° (range, −2.9–10.5°). One patient had shortening.

No nonunion, broken plates, or implant failures were observed. Three patients had malunion. No revision operations were required during the study period.

In patients with osteoporosis (BMD < −2.5), the JLETS was significantly reduced. However, there were no statistically significant differences in the other factors between osteoporotic and normal patients (Table 2).

**Table 2:** Comparison between osteoporosis patients and normal patients

Patient parameter	BMD < −2.5 (N = 5)	BMD ≥ −2.5 (N = 13)	Total (N = 18)	***P***
**Mean age, (years)**	75.4 ± 7.0	74.6 ± 5.7	74.8 ± 5.9 (68–89)	0.809
**Bone mineral density(T-score)**	−3.5 ± 0.48	−1.4 ± 0.8	−2.0 ± 1.2 (−4.3–0.3)	< 0.001
**Time to bone union (weeks)**	15.8 ± 4.6	19.5 ± 11.1	18.4 ± 9.8 (10–51)	0.493
**JLETS**^a^	29.6 ± 4.0	40.7 ± 7.7	37.6 ± 8.5 (24–53)	0.008
**Varus-valgus angle (degrees)**	4.4 ± 3.5	2.7 ± 3.2	3.2 ± 3.3 (−2.9–10.5)	0.347
**Shortening, n (%)**				0.722^b^
**Yes**	0 (0.0)	1 (7.7)	1 (5.6)	
**No**	5 (4.7)	12 (92.3)	17 (94.4)	
**Malunion, n (%)**				0.650^b^
**Yes**	1 (20.0)	2 (15.4)	3 (16.7)	
**No**	4 (80.0)	11 (84.6)	15 (83.3)	

Note: Data presented as mean ± standard deviation unless otherwise indicated

^a^Jeju Lower Extremity Trauma Scale (the modified WOMAC), ^b^Fisher exact test

In addition to the correlation between BMD and JLETS, bone union took longer in older patients (Table 3).

**Table 3:** Evaluation of correlation between other factors

	Age	BMD	Time to union	JLETS	Varus-valgus	Shortening
Age	1.000					
BMD	−0.293	1.000				
Time to union	0.489*	0.151	1.000			
JLETS	−0.206	0.489*	−0.076	1.000		
Varus-valgus	0.107	−0.419	0.019	−0.076	1.000	
Shortening	−0.114	−0.186	−0.449	−0.208	−0.084	1.000

Spearman’s rho correlation test: *P < 0.05

## Discussion

In this study, we evaluated the effects of MIPO with dual plating to treat periprosthetic distal femoral fractures following TKA in 18 patients and found that MIPO with dual LCP is a reliable method for stabilizing periprosthetic distal femoral fractures following TKA, with satisfactory bone union rates and low complication rates.

Recent reports focusing on retrograde IM nailing and LCP plating support the priority of these two procedures over conventional plating [8, 10, 15, 18]. The comparison of LCP plating and retrograde IM nailing showed no statistically significant differences with respect to nonunion rates. LCP plating showed significantly lower malunion rates than retrograde IM nailing in a systematic review [19], and the authors proposed three reasons for the superiority of the LCP over retrograde IM nailing for malunion rates. First, the starting point for retrograde IM nailing is dictated by the position of the femoral component and can cause malreduction. Second, retrograde IM nailing does not have the capacity to fill the wide metaphyseal intramedullary space, which allows for potential movement of the distal fragment relative to the nail. Third, the LCP plate offers more distal fragment fixation options than retrograde IM nailing.

The recently introduced dual plating technique with medial plate application for extremely distal periprosthetic fractures can provide sufficient stability [13]. In a biomechanical study, dual plating was more effective and had greater stability than simple lateral plating [14]. Medial plating is not widely attempted because of potential injury to the femoral artery. However, in a recent study, a medial plate could be safely applied on the anteromedial aspect of the distal femur to a distance of up to 8 cm distal to the lesser trochanter [20]. A recent cadaveric study has stated that the distal 60% of the femur is a safe zone for medial plating [21]. Hence, medial plating along with MIPO and lateral LCP plating is not only safe but also rigid enough to support a medial-sided fracture.

In a previous study that used double plating in different directions (orthogonal, i.e., lateral and anterior) [22], various types of periprosthetic fractures (around total hip replacement arthroplasty, TKA, and inter-prosthetic fractures) were assessed, while including only one case of periprosthetic distal femoral fracture following TKA. PHILOS plates have many advantages in medial fixation, including a similar shape to the contour of the medial condyle and a size that does not interfere with the femoral component of TKA. A previously reported limitation of medial plates is that they exert a fixing force mainly in the coronal plane. However, the fixing direction of the screw in the PHILOS plate is from the anteromedial to posterolateral direction, and this fixation force on the sagittal plane is additionally applied to the distal femur, which contributes to additional stabilization in the coronal plane and introduces new stability in the sagittal plane. This diagonal plating has two vectors applied in the coronal and sagittal planes. Additionally, with the use of the PHILOS plate, the application of many screws with various angles is possible and provides a rigid and stable fixation.

No direct evidence exists that malunited periprosthetic fractures post-TKA is associated with early component failure or wear; however, previous literature suggests that component malposition could cause such complications [23]. Malunion of these fractures also causes component malposition and hence may be related to component failure or wear. This can be resolved by MIPO with an LCP placed laterally and initial fracture fixation with a PHILOS plate medially for periprosthetic distal femoral fractures. Moreover, double plating with an anteromedial PHILOS plate offers additional stabilization in the coronal plane and introduces stability in the sagittal plane. Hence, this technique could potentially reduce TKA component failure or wear after periprosthetic distal femur fractures.

Despite satisfactory outcomes, our study has some limitations. First, we included a small number of cases, as periprosthetic distal femoral fractures have a low incidence. Our small sample size may increase the possibility of incomplete documentation and the risk to miss significances. Second, this was a retrospective study with no control group; therefore, our results should be validated by larger studies in the future. A long-term prospective randomized control study with a larger scale is necessary to further evaluate the efficacy of the dual plate technique compared with a conventional single plate fixation. Such would aid in strengthening the significance of each sample group by implementing random allocation of each type of fixation method.

## Conclusion

When treating technically difficult periprosthetic distal femur fractures following TKA, using a dual plating technique with a distal femur LCP laterally and a PHILOS plate medially increases the accuracy of reduction and stability at the fracture site. Anteromedial PHILOS plates can offer additive stabilization in both coronal and sagittal planes.

## Data Availability

The datasets used and/or analyzed during the current study are available from the corresponding author on reasonable request.

## Abbreviations

TKA: Total knee arthroplasty
MIPO: Minimally invasive plate osteosynthesis
LCP: Locking compression plate
PHILOS: Proximal humeral internal locking system
IM: Intramedullary
ROM: Range of motion
BMD: Bone mineral density
WOMAC: Western Ontario McMaster Universities Index of Osteoarthritis
JLETS: Jeju Lower Extremity Trauma Scale
VAS: Visual analog scale
AP: Anteroposterior
K-wires: Kirshner wires
CPM: Continuous passive motion
